# Comparative transcript profiling explores differentially expressed genes associated with sexual phenotype in kiwifruit

**DOI:** 10.1371/journal.pone.0180542

**Published:** 2017-07-03

**Authors:** Ping Tang, Qiong Zhang, Xiaohong Yao

**Affiliations:** Key Laboratory of Plant Germplasm Enhancement and Speciality Agriculture, Wuhan Botanical Garden, the Chinese Academy of Sciences, Wuhan, Hubei, China; Henan Agricultural University, CHINA

## Abstract

**Background:**

Kiwifruit is a perennial, deciduous and functionally dioecious plant. However, very little is known about the whole-genome molecular mechanisms contributing to distinct sexual phenotypes. To gain a global view of genes differentially expressed between male and female flowers, we analyzed genome-wide gene expression profiles in the flowers of male and female plants using high-throughput RNA sequencing.

**Results:**

A total of 53.5 million reads were generated. Based on the alignments of unigenes to kiwifruit genome predicted genes, a total of 39,040 unique genes with a mean length of 970 bp were identified. There were 2,503 UniGenes differentially expressed between female and male flowers, with 1,793 up-regulated and 710 down-regulated in the female flowers. Moreover, the gene expression pattern of 17 out of 19 unigenes differentially expressed between male and female flowers revealed by RNA-Seq was confirmed by real-time quantitative PCR (qRT-PCR).

**Conclusions:**

Here, we obtained a large number of EST sequences from female and male flowers of kiwifruit. This comparative transcriptome analysis provides an invaluable resource for gene expression, genomics, and functional genomic studies in *A*. *chinensis* and its related species. This study also represents a first step toward the investigation of genes involved in kiwifruit sex determination.

## Introduction

The genus *Actinidia*, commonly known as kiwifruit, contains 54 species and 21 botanical varieties, and most of these species are native to China [1,2]. Kiwifruit has a delicious flavor and high nutritional value and is especially rich in ascorbic acid (vitamin C), minerals and dietary fiber. Kiwifruit is an important horticultural crop with a short history of domestication (approximately 100 years), and was first cultivated in New Zealand and subsequently in other countries such as Chile, China and Italy [1]. Currently, numerous varieties of *A*. *chinensis* have been developed through selection from wild populations and hybridization breeding [3]. According to data from the Food and Agriculture Organization, approximately 3.1 million metric tons of fresh kiwifruit were produced in 2014 (http://faostat.fao.org). In China, the current annual growing area is over 0.24 million hectares, with an annual production of 1.8 million metric tons [4].

All known *Actinidia* taxa appear to be functionally dioecious plant whose flowers are morphologically hermaphrodite yet functionally unisexual. Females bear flowers with a functional ovary but produce non-viable pollen, whereas male flowers shed viable pollen but have no functional ovaries [5,6]. Pollen development in pistillate flowers or pistil growth in staminate flowers stops at the late phase of flower bud development [7,8]. *Actinidia* has an active Y sex determination system (XnX/XnY) where male plants contain Y chromosomes [5,9,10]. However, hermaphroditic flowers or monoecious plants are occasionally produced in *Actinidia* [1], suggesting it arose during the early stages of sex chromosome evolution [11]. The sex-linkage group has been identified and the sex-determining region is located on chromosome 25 in the genus *Actinidia* [10,12]. The molecular mechanisms of sex determination have been intensively studied in the model plants *Silene* [13], cucumis [14], papaya [15] and strawberry [16]. The elucidation of sex-determining mechanisms is important for kiwifruit breeding practices. Although several sex-related makers have been identified in kiwifruit [5,6,12], few studies have focused on the molecular mechanisms underlying *Actinidia* sex determination.

Great advances have been made in developing genomic resources for *A*. *chinensis*, including expressed sequence tag (EST) [17], genetic linkage maps [12], and whole genome sequences [18]. Kiwifruit has a relatively small genome (~750 Mb), with a published genome assembly of approximately 81.3% of its estimated genome size [18]. The number of available kiwifruit transcriptomes is increasing rapidly in NCBI databases [17,19]. Identifying differentially expressed genes (DEGs) between different sex phenotypes is an essential step in elucidating the mechanisms of sex determination. Currently, high-throughput RNA sequencing (RNA-Seq) is the most useful tool for discovering genes with both low- and high-level gene expression, and this technology can facilitate the identification of gene expression and regulatory mechanisms. In this study, global gene transcriptional levels in both male and female kiwifruit flowers were examined using the Illumina HiSeq^™^ 2000 sequencing platform to reveal the molecular mechanisms of sex determination. To our knowledge, this study is the first to undertake genome-wide comparative analysis of gene expression in young floral buds between male and female kiwifruit plants. The transcriptome data generated here will serve as a valuable resource for novel gene discovery, laying a sound foundation for elucidating the sex-determining mechanisms of *A*. *chinensis*.

## Results

### Transcriptome assembly and annotation

A total of 53,545,356 raw reads from the two libraries were generated. After removing adaptor sequences, ambiguous reads and low-quality reads, a total of 49,069,919 clean reads with an average length of 200 bp were obtained. Among these, 24,766,866 were from female flowers and 24,303,053 were from male flowers (Table 1). All clean reads are available from the NCBI Short Read Archive (SRA) database under accession number SRR5650770. All clean reads were assembled into 87,433 contigs with a mean length of 543 bp. Clean reads were then aligned to the kiwifruit reference genome database. Approximately 51.5% of unigenes could be mapped to the reference genome, allowing 95% sequence identity and 80% length coverage. This limited percentage of matching sequences can likely be ascribed to different tissues of origin and incomplete genome coverage. A total of 39,040 non-redundant unigenes with a mean length of 970 bp were obtained. The GC content for transcript sequences of the male flowers and female flowers was 41.3% and 41.2%, respectively.

**Table 1:** Summary of sequence analysis.

	Female flowers	Male flowers	Total
Total number of raw reads	26,516,018	27,029,338	53,545,356
Total number of clean reads	24,766,866	24,303,053	49,069,919
Average read length	200 bp	200 bp	200 bp
Total mapped reads (percent of clean reads)	16,964,593 (68.5%)	25,360,525 (51.8%)	29,552,468 (59.6%)
Unique match (percent of clean reads)	9,584,130 (38.7%)	7,974,472 (32.8%)	17,558,602 (35.8%)
Mutliple match (percent of clean reads)	7,380,463 (29.8%)	4,613,403 (19.0%)	11,993,866 (24.4%)
Total number of unigenes (≥ 100 bp)	/	/	39,040
Mean length of unigenes	/	/	970

Note: Unique match: the number of reads mapping to the unique location of the reference sequence; Multiple match: the number of reads mapping to multiple locations of the reference sequence

### Comparison of female and male flower transcriptomes

Differential expression analysis identified 2,503 genes differentially expressed in male and female flowers with *p* < 0.001 (S1 Table), and many more genes were over-expressed in female flowers (1,793) compared to male flowers (710). Twenty transcription factors with significantly higher expression in female flowers and 64 with significantly higher expression in male flowers were identified (Table 2 and S2 Table). The MYC transcription factor family contained 2 over-represented members in female flowers and 14 over-represented members in male flowers (Table 2), suggesting these genes might play important roles in sex determination. MYB family transcription factors are well known to be involved in responses to hormones and flower development. In the present study, 6 and 8 over-expressed MYB family members were detected in female and male flowers, respectively. Moreover, other transcription factor families such as AP2/ERF and bZIP were over-represented in male flowers, indicating that these proteins might play important roles in regulating *A*. *chinensis* sex determination (Table 2).

**Table 2:** Distribution of over-expressed transcription factors in female and male flowers.

Transcription factor family	Female flowers	Male flowers
MYC	2	14
MYB	6	8
MYB-like	0	1
MADS-box	1	1
bZIP	0	6
GRAS	0	3
SBP-box	0	2
bHLH25	1	0
TCP	1	2
GATA	0	2
AP2/ERF	0	12
WRKY	1	3
other	8	10
Total	20	64

**Fig 1:**
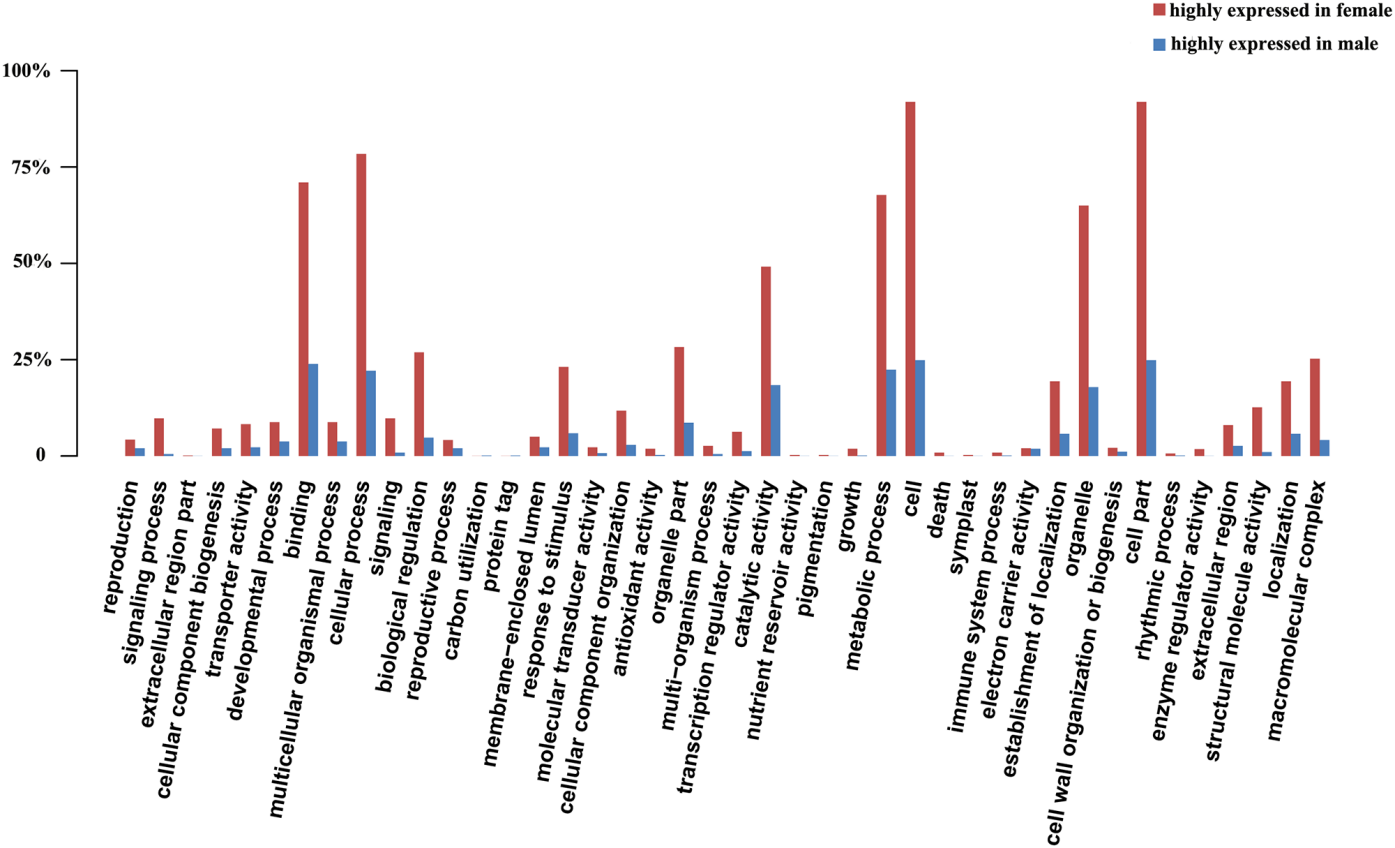
GO analysis for genes differentially expressed between female and male flowers.

**Fig 2:**
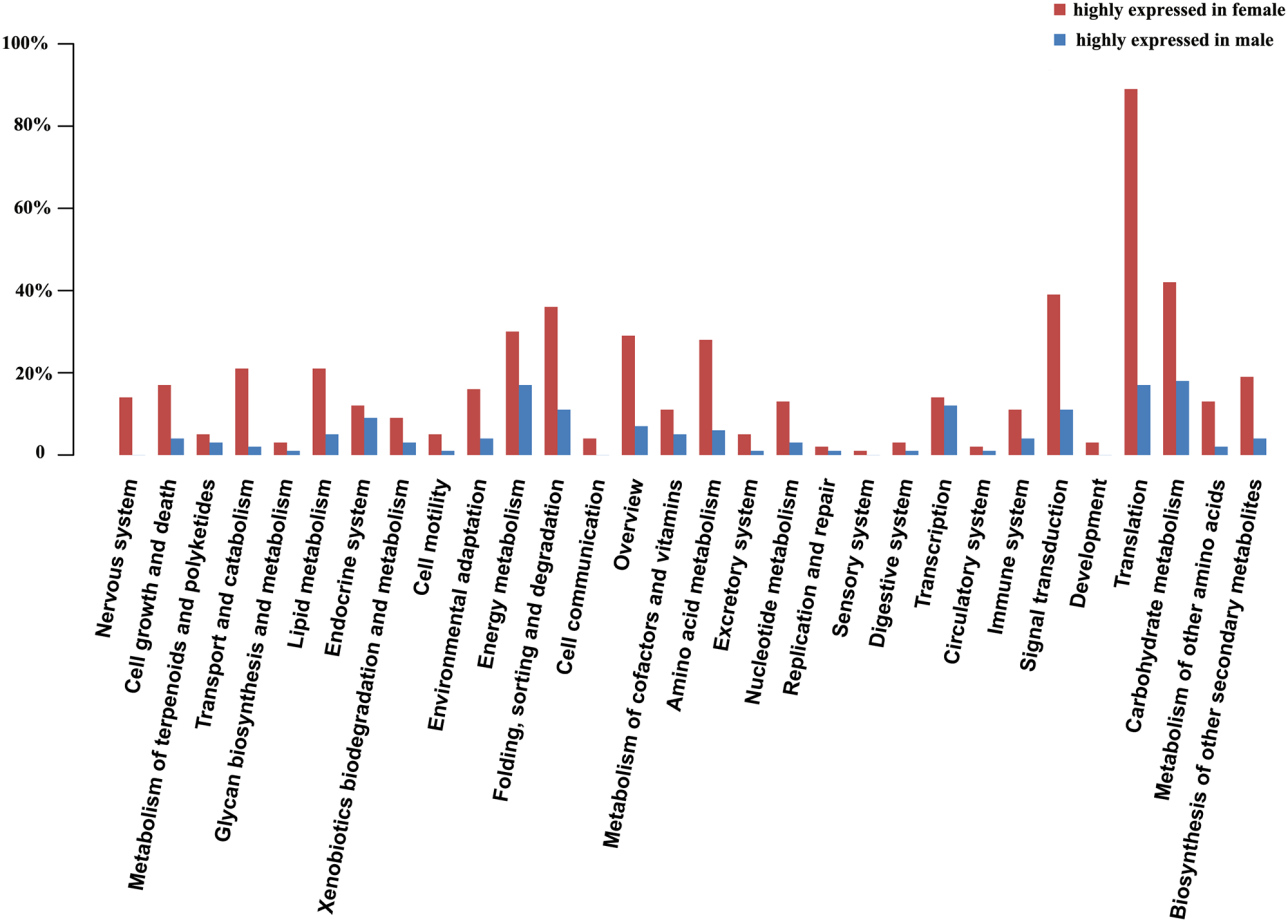
KEGG analysis for genes differentially expressed between female and male flowers.

**Fig 3:**
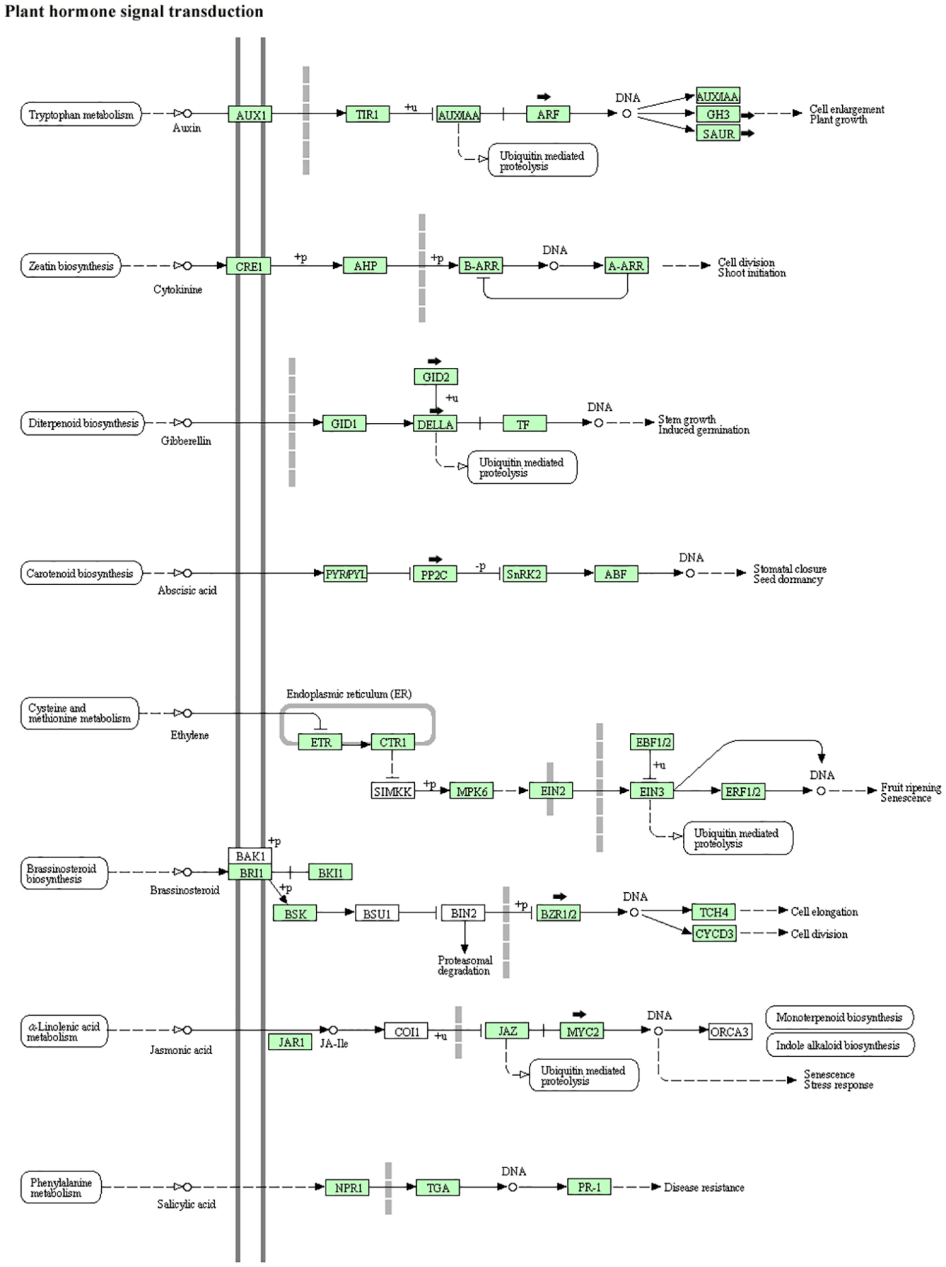
Plant hormone signal transduction. Thirty-seven unigenes were assigned to plant hormone signal transduction pathways by KEGG. The kiwifruit unigenes involved in these pathways are marked in green. Arrows indicate differentially expressed genes closely associated with hormone synthesis and metabolism.

We performed GO enrichment analysis using Blast2Go to further characterize differentially expressed genes. This procedure revealed that up- and down-regulated DEGs were mainly involved in cell part, cell, cellular process, binding, metabolic process, catalytic activity, organelle part and biological regulation (Fig 1). To further elucidate the biological functions of DEGs, we performed KEGG pathway analysis. Based on annotations of the unigenes, a biochemical pathway database containing 336 predicted pathways was created. Translation, carbohydrate metabolism, signal transduction, protein folding, sorting and degradation, and energy metabolism were identified as over-represented pathways corresponding to DEGs (Fig 2). In addition, a total of 37 unigenes were assigned to plant hormone signal transduction pathways by KEGG (Fig 3).

### Verification of RNA-Seq data by real-time quantitative RT-PCR

**Fig 4:**
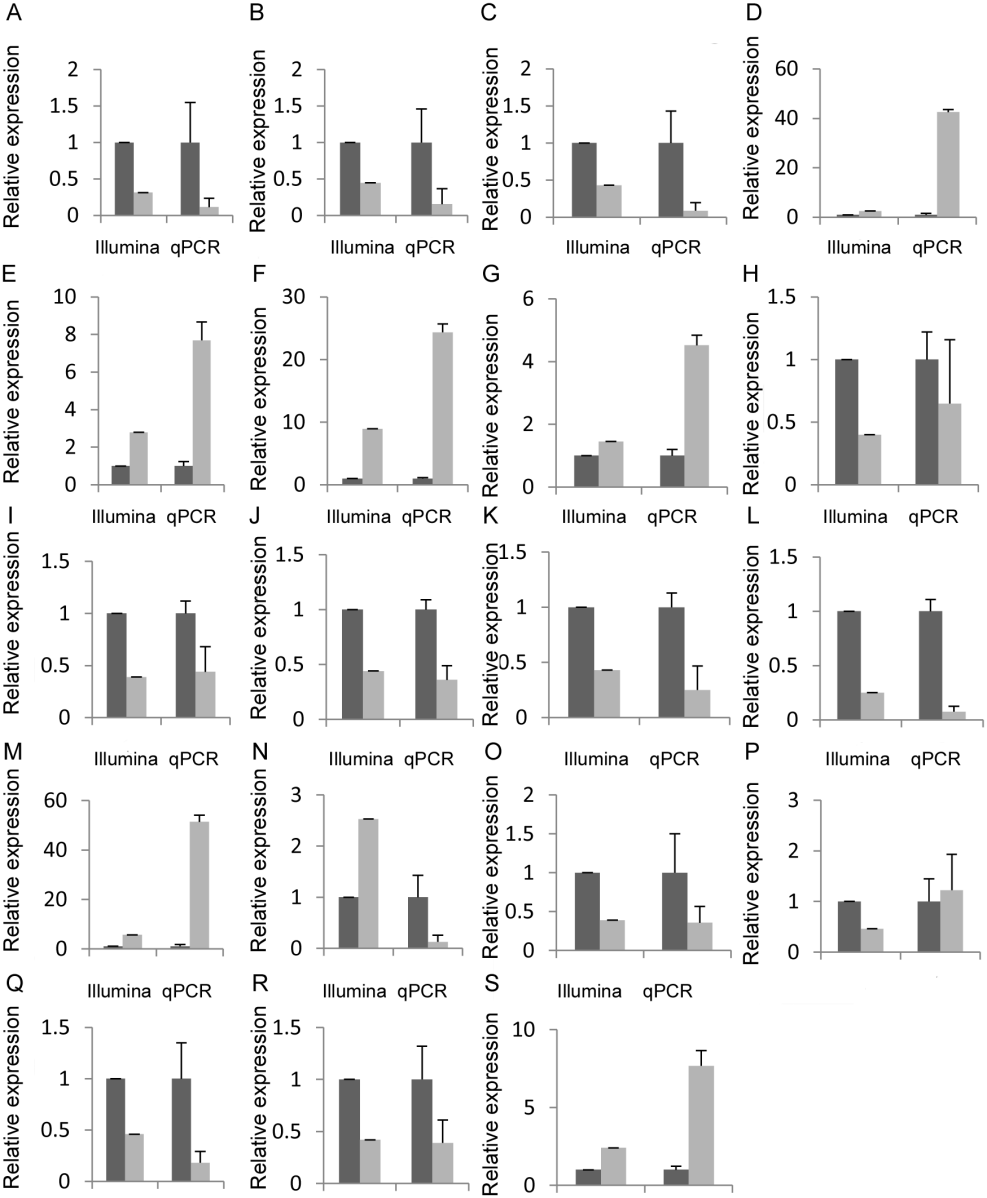
Validation of 19 genes differentially expressed between female and male flowers using qRT-PCR. These differentially expressed genes encode V-type proton ATPase subunit E (A), DELLA protein GAI (B), Putative leucine-rich repeat receptor-like protein kinase family protein (C), Putative uncharacterized protein OSJNBb0014M19.23–2 (D), Male sterility MS5 (E), Tapetum-specific protein A9 (F), Pollen ole e 1 allergen and extensin family protein (G), Flowering promoting factor-like protein (H), Transcription factor MYC (I), WRKY transcription factor (J), Transcription factor MYC2 (K), Pistil-specific extensin-like protein (L), Unknown protein (M), MYB transcription factor (N), Mitochondrial phosphate carrier protein (O), Nudix hydrolase 2 (P), Ethylene responsive transcription factor 12 (Q), Ubiquitin carboxyl-terminal hydrolase (R) and Small ubiquitin-related modifier 2 (S).

To confirm the expression patterns of a subset of differentially expressed genes identified by Illumina sequencing, nineteen genes were randomly selected for real-time RT-PCR analysis (Fig 4). Seventeen of these were validated by qRT-PCR. V-type proton ATPase subunit E, DELLA protein GAI, putative leucine-rich repeat receptor-like protein kinase family protein, flowering promoting factor-like protein, transcription factor MYC, WRKY transcription factor, transcription factor MYC2, pistil-specific extensin-like protein, mitochondrial phosphate carrier protein, ethylene responsive transcription factor 12 and ubiquitin carboxyl-terminal hydrolase were significantly up-regulated in female flowers as expected based on DGE analyses. Putative uncharacterized protein OSJNBb0014M19.23–2, male sterility MS5, tapetum-specific protein A9, pollen ole e 1 allergen and extensin family protein, unknown protein and small ubiquitin-related modifier 2 were significantly up-regulated in male flowers, which was consistent with the Illumina sequencing results. MYB transcription factor and Nudix hydrolase 2 could not be confirmed by qRT-PCR. Further studies will be needed to better elucidate the inconsistent expression patterns between RNA sequence and the qRT-PCR analysis for these two genes.

## Discussion

Currently, several major sex-determining genes have been detected and characterized in various plants including cucurbit, maize and other species [20,21]. However, for kiwifruit, the mechanisms of sex determination remain largely unknown. High-throughput RNA-seq has been widely used to discover novel genes and investigate gene expression profiles [22]. In the present study, we used the NGS technology RNA-seq to identify genes associated with sex determination by comparing the gene expression profiles between female and male kiwifruit flowers (comparative transcriptome approach). The ESTs developed in the present study represent a significant addition to the existing kiwifruit genomic resources and provide important insights into the mechanisms of sex determination in kiwifruit. For example, a large number of unigenes involved in hormone biosynthesis, membrane transport and gene transcription showed differential expression patterns in the present study.

DEGs involved in pollen wall assembly, including genes encoding chalcone synthase, expansins, beta-1,3-glucanase, pollen Ole e 1 family allergen and extension, were significantly up-regulated in male flowers (S1 Table). As a key enzyme in the flavonoid biosynthesis pathway (S1 Fig), chalcone synthase is well known for its role in male fertility [23]. Previous studies have shown that beta-1,3-glucanase performs various functions related to male gametophyte development and pollination [24,25]. In addition, some genes involved in cell wall loosening were prominently expressed in male flowers. In this study, genes encoding polygalacturonase (PG), pectate lyase and endoglucanase were annotated in our DEG data. Transcriptional analysis of watermelon has revealed that PG and pectate lyase are widely involved in the pollen development process [25].

Plant growth molecules such as hormones play important roles in various processes related to plant development, including cell elongation, flower development, sex determination and sex differentiation, among others [21]. In the present study, genes associated with hormone synthesis and metabolism showed significant down-regulation or up-regulation between male and female flowers (Fig 3). Ethylene is a major hormone for regulating sex determination and promotes female development in melon and cucumber [26]. In our study, thirteen ethylene-responsive transcription factors were up-regulated in female flowers. Auxin has been shown to play a critical role in inducing femaleness through its stimulation of ethylene production [27,28], and we show here that three auxin-induced transcripts (Achn310311, Achn033901, Achn340641) have higher expression in female kiwifruit flowers.

The regulation of cell elongation and plant growth by gibberellic acid (GA) is becoming increasingly elucidated [21,28], and the promotion of female flower development by GA biosynthesis or perception has been observed in maize [29]. In the present study, a transcription factor (Achn128851) encoding a member of the DELLA protein family, an ortholog of the Gibberellin Insensitive (GAI) gene in Arabidopsis [30], had higher expression in female flowers. Brassinosteroids (BRs) are also known for their role in inducing femaleness in cucumber through inducing production of ethylene [31]. BRs are involved in ethylene synthesis and so their down-regulation can lead to reduced ethylene production. Here, we identified a gene (Achn353131) belonging to the BZR1-BES1 family with higher expression in female flowers. However, it has been reported that BR also promotes male flower development. Thus, BR may be not the decisive factor for sex determination [32].

Polyamines (PAs) have been reported to participate in plant reproduction, especially pollen development and germination [33]. Specifically, PAs play roles in normal pollen development and growth in *Malus domestica* [34] and *Nicotiana tabacum* [35]. Our analysis identified a polyamine oxidase gene (Achn112371) that has higher expression in male flowers. Consistent with this, the role of PA in pollen development and germination has been previously demonstrated in kiwifruit [33].

Transcription factors are well known to control plant growth and development as well as hormone and stress responses [36]. Several transcription factors have been reported to be specifically related to the plant sex determination. For example, a C2H2 zinc finger transcription factor (CmWIP1) plays a central role in the development of unisexual male flowers of cucumber [37]. In our study, a MYC family transcription factor (Achn136071) showed higher expression in male flowers, which is consistent with a previous report linking a MYC transcription factor (DA) to sex determination in *Drosophila* [38]. However, in contrast to animals, the role of MYC transcription factors in plant sex determination is currently unexplored and requires further study [28]. In addition, a MADS-box transcription factor controlling the specification of stamen primordia was found in our DEGs database [39].

Other putative transcription factors identified in this study, such as bZIP protein, bHLH protein, WRKY DNA-binding protein, and NAC domain proteins, have been previously documented to play important roles in various processes of plant development. For instance, bZIP transcription factors are thought to regulate diverse biological processes including pathogen defense, light and stress signaling, seed maturation and flower development [21,28]. However, an association of these transcription factors with plant sex determination has not been documented. More broadly, our study has uncovered a large number of genes differentially expressed between female and male flowers that have not been previously associated with sex differentiation. Hence, further studies are needed to reveal whether these genes are functionally related to the process of kiwifruit sex determination.

## Materials and methods

### Plant materials

**Fig 5:**
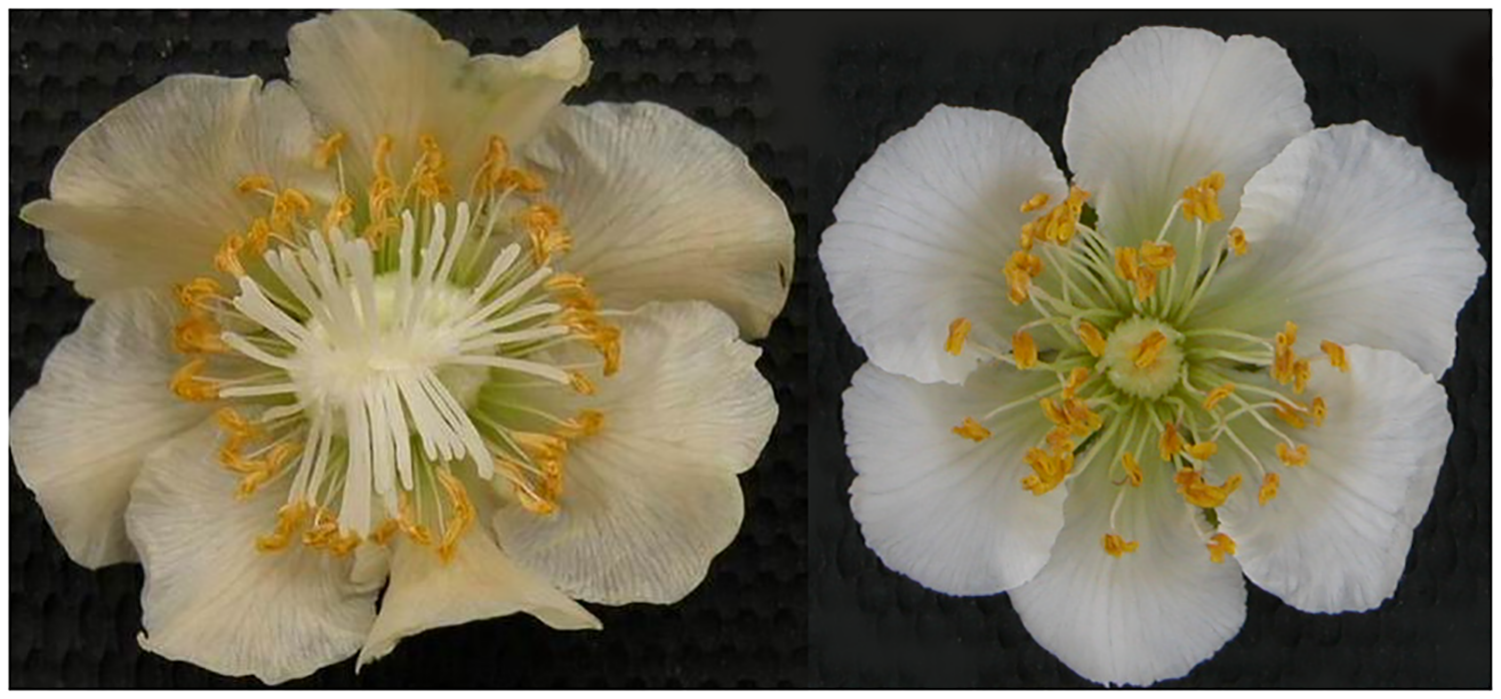
Morphological differences in the floral organs of female and male kiwifruit individuals.

The female plant was ‘Hongyang’, and the male plant was derived from F_1_ hybrids between ‘Hongyang’ (♀) and ‘Guihai No. 4’ (♂), both of which were cultivated at the National Actinidia Germplasm of Wuhan Botanical Garden, Chinese Academy of Science, Wuhan, China (Fig 5). In April 2014, twenty young floral buds from three female and male individuals (the same genotype) were sampled and pooled together. All samples were immediately frozen separately in liquid nitrogen and stored at -80°C prior to RNA extraction.

### RNA isolation, cDNA library construction and sequencing

Total RNAs were isolated from young floral buds using Trizol Reagent. mRNA was purified with the RNA Clean-up Kit according to the manufacturer’s instructions. The purified RNA was subsequently fragmented into ~200 bp pieces by treatment with divalent cations at 75°C. First-strand cDNA synthesis was performed from the cleaved RNA fragments using reverse transcriptase (Invitrogen) with random hexamer primers, and second-strand cDNA was synthesized using RNase H and DNA polymerase I (New England BioLabs). Second generation sequencing was performed on the Illumina HiSeq 2000 instrument from Shanghai Haiyu Biotechnology Co., Ltd.

### Assembly and annotation

Before assembly, raw reads were filtered to remove adaptor sequences, low-quality reads and reads containing poly-N. Transcriptome de novo assembly of clean reads was performed using Trinity software [40]. All assembled unigenes were then mapped to the kiwifruit genome database (http://bioinfo.bti.cornell.edu/cgi-bin/kiwi) using Bowtie 2 software [41] with the default parameters.

### Quantification of gene expression levels and differential expression analysis

The differentially expressed genes between the two samples were identified using the MARS model in DEGseq R package (1.12.0). Corrected *p*-value < 0.001 and an absolute value of the log2 ratio > 1 were used as thresholds to determine the significant difference in gene expression between the two samples. GO terms enriched in the set of differentially expressed genes were implemented using the GO:TermFinder [42]. Moreover, functional classifications were conducted using Blastall software against the Kyoto Encyclopedia of Genes and Genomes (KEGG) database. KEGG pathways in DEGs with a q-value ≤ 0.05 are considered as significantly enriched.

### qRT-PCR analysis

The expression patterns of nineteen randomly selected genes potentially involved in sex determination were identified by real-time quantitative RT-PCR (qRT-PCR). The primers for qRT-PCR were designed using Primer3 software version 0.4.0 (http://frodo.wi.mit.edu/primer3/) (S3 Table). Three biological replicates for each sample were performed on a Bio-Rad iQ5 Optical System Real Time PCR System (Bio-Rad, USA) using a SYBR Green based PCR assay. The thermal cycle conditions were as follows: 95°C for 3 min followed by 40 cycles of 30 s at 95°C, 20 s at 58°C, and 15 s at 72°C. With the β-Actin gene and elongation factor gene as the internal reference genes, the relative expression levels were analyzed using the 2^-△△CT^ method [43].

## Supporting information

S1 Fig Flavonoid biosynthesis pathway. Thirty-four unigenes were assigned to the flavonoid biosynthesis pathway by KEGG. The kiwifruit unigenes involved in these pathways are marked in green.
(TIF)

S1 Table Differentially expressed genes between female and male flowers. (XLSX)

S2 Table The detailed fold changes of transcription factors between female and male flowers. (XLSX)

S3 Table Primer sequences used for qRT-PCR. (XLSX)
