# Supplementary figures and images for: Comparative transcript profiling explores differentially expressed genes associated with sexual phenotype in kiwifruit
Source: PLoS One. 2017 Jul 3;12(7):e0180542. doi: 10.1371/journal.pone.0180542 (PMC5495465; doi:10.1371/journal.pone.0180542)

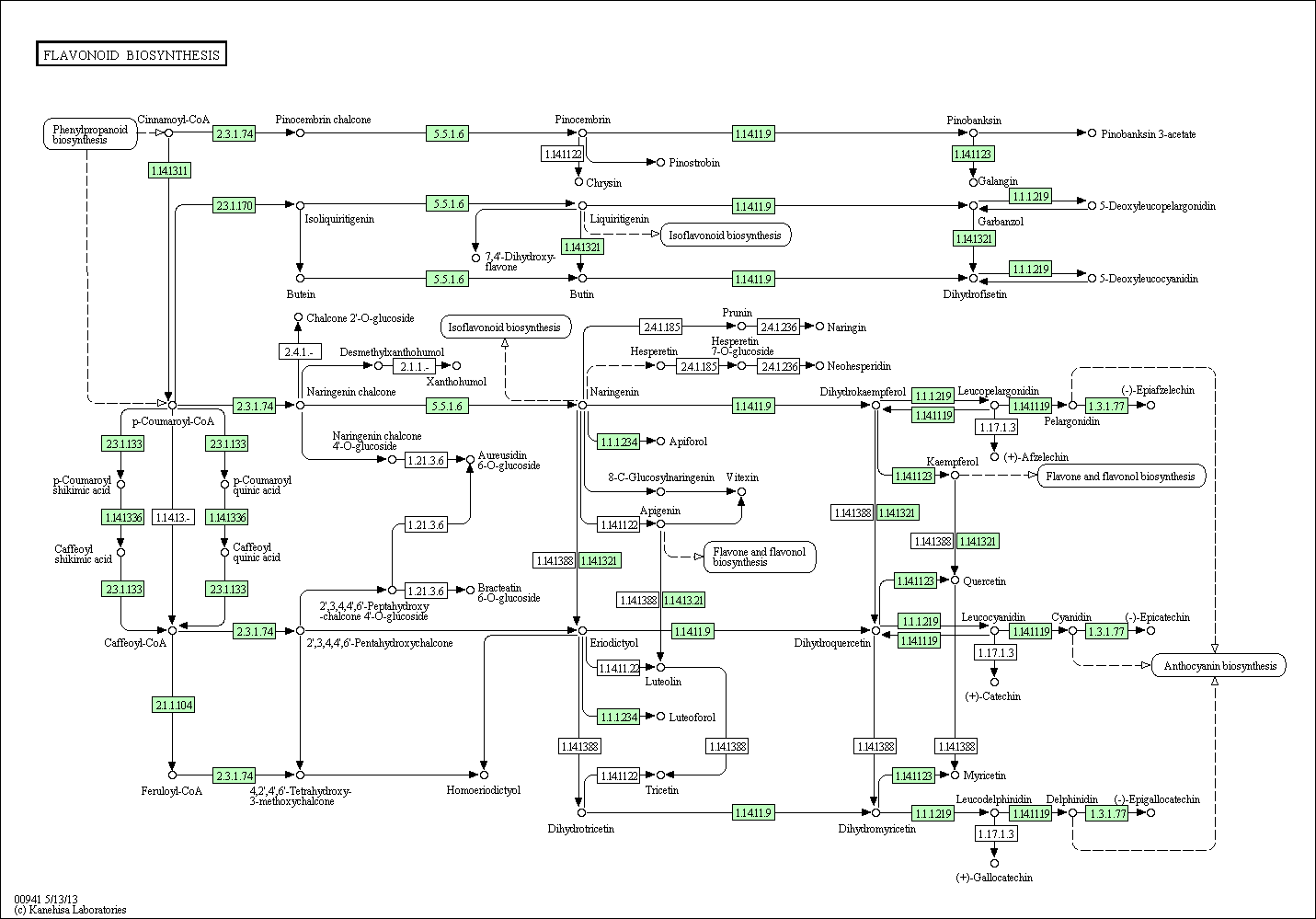

Supplement: S1 Fig — Thirty-four unigenes were assigned to the flavonoid biosynthesis pathway by KEGG. The kiwifruit unigenes involved in these pathways are marked in green. (TIF) [file pone.0180542.s001.tif]
